# Reduced Graphene Oxide-Based Silver Nanoparticle-Containing Composite Hydrogel as Highly Efficient Dye Catalysts for Wastewater Treatment

**DOI:** 10.1038/srep11873

**Published:** 2015-07-17

**Authors:** Tifeng Jiao, Haiying Guo, Qingrui Zhang, Qiuming Peng, Yongfu Tang, Xuehai Yan, Bingbing Li

**Affiliations:** 1 State Key Laboratory of Metastable Materials Science and Technology, Yanshan University, Qinhuangdao 066004, P. R. China; 2 Hebei Key Laboratory of Applied Chemistry, School of Environmental and Chemical Engineering, Yanshan University, Qinhuangdao 066004, P. R. China; 3 National Key Laboratory of Biochemical Engineering, Institute of Process Engineering, Chinese Academy of Sciences, Beijing 100190, P. R. China; 4 Department of Chemistry and Biochemistry, Science of Advanced Materials Doctoral Program, Central Michigan University, Mount Pleasant, MI 48859, USA

## Abstract

New reduced graphene oxide-based silver nanoparticle-containing composite hydrogels were successfully prepared *in situ* through the simultaneous reduction of GO and noble metal precursors within the GO gel matrix. The as-formed hydrogels are composed of a network structure of cross-linked nanosheets. The reported method is based on the *in situ* co-reduction of GO and silver acetate within the hydrogel matrix to form RGO-based composite gel. The stabilization of silver nanoparticles was also achieved simultaneously within the gel composite system. The as-formed silver nanoparticles were found to be homogeneously and uniformly dispersed on the surface of the RGO nanosheets within the composite gel. More importantly, this RGO-based silver nanoparticle-containing composite hydrogel matrix acts as a potential catalyst for removing organic dye pollutants from an aqueous environment. Interestingly, the as-prepared catalytic composite matrix structure can be conveniently separated from an aqueous environment after the reaction, suggesting the potentially large-scale applications of the reduced graphene oxide-based nanoparticle-containing composite hydrogels for organic dye removal and wastewater treatment.

In recent years, harmful chemical compounds have become the main cause of water pollution. For instance, organic dyes are often discharged with wastewater into the local environment without adequate treatment. Rapid and convenient removal of organic dyes from wastewater has been a challenging issue faced by scientists^1^,^2^,^3^,^4^. For example, O. Akhavan *et al.* successfully reported the preparation and magnetic separation application of superparamagnetic ZnFe_2_O_4_/reduced graphene oxide (rGO) composites by hydrothermal reaction method^5^. In addition, their group has also investigated some bacteria bioactivity and interaction with environment by aggregated graphene nanosheets as encapsulating material and effective photothermal agent^6^. On the other hand, as an attractive class of soft material, there is considerable interest in assembling graphene and its derivatives into 3D porous gels with ultralow density, high compressibility, high conductivity, large surface areas and strong mechanical strength^7^,^8^,^9^,^10^,^11^,^12^,^13^,^14^. In addition, the GO-based hydrogels have been obtained by adding polymers, macromolecules, small organic molecules, or cation compounds into the aqueous dispersion of GO^15^,^16^,^17^,^18^. In the process of self-assembly, the main driving forces for gelation are hydrogen bonding, π-π interaction, or electrostatic interaction. Considering the porous structure and the excellent mechanical strength from the strong π–π stacking of graphene sheet and its derivatives, the 3D gels could be an ideal support for immobilizing various nanoparticles, which can theoretically provide large water/solid interfaces, where organic dyes can be easily accessed because of the interconnected porous structure. Although the preparation of graphene-based composite hydrogels for photocatalyst has been previously reported^19^,^20^,^21^,^22^, recovering the photocatalyst after waste water purification has been a challenging issue. Thus, polymer-based photocatalyst nanocomposites have been attracting more interest due to their advantages for photocatalyst recovery. Among many templates, microporous/mesoporous GO hydrogel networks, are particularly appropriate for the *in situ* production of nanoparticle compared to the conventional non-aqueous synthetic approaches^23^,^24^,^25^,^26^,^27^,^28^,^29^,^30^. For example, Gao *et al.* reported the preparation of a nanocomposite graphene hydrogel by employing vitamin C to attain a supramolecular 3D network of hybrid nanostructured materials^30^.

In this work, we demonstrate the formation GO-based hydrogels in the presence of polyethyleneimine (PEI) and the *in situ* formation of RGO based silver nanoparticles-containing composite hydrogel using an *in situ* reduction approach. Here, silver ions and GO are simultaneously reduced by using vitamin C in a single step within the hydrogel phase in this reported method to form a nanocomposite system. The as-prepared silver nanoparticles homogeneously and uniformly modified the surface of the RGO nanosheets and the organized nanocomposite structures were successfully achieved. Interestingly, this composite hydrogel matrix can be utilized as highly efficient catalyst for wastewater treatment.

## Results and Discussion

**Figure 1:**
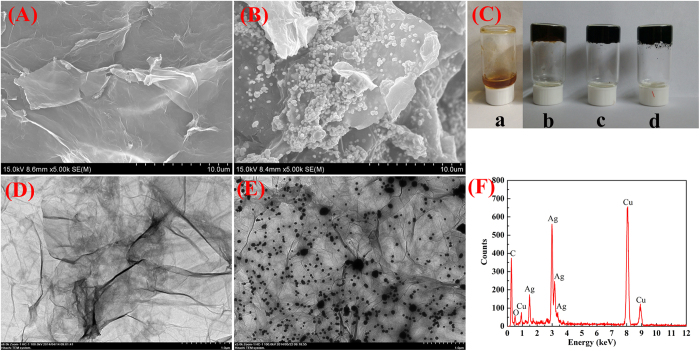
SEM and TEM images for the lyophilized RGO/PEI hydrogel (**A**, **D**) and RGO/PEI/Ag hydrogel (**B**, **E**). **C** is Photographs: a, GO aqueous solution; b, GO/PEI hydrogel; c, RGO/PEI hydrogels; d, RGO/PEI/Ag hydrogel. F is EDXS taken on the RGO/PEI/Ag hydrogel shown in **E**.

Here, the reduced graphene oxide (RGO)-based hydrogel was obtained by the *in situ* reduction of GO using vitamin C in the presence of heat (90 °C). Vitamin C acts as a nontoxic, environmentally friendly reducing agent and the entire reduction process does not produce any gaseous by-products. RGO-based gels have a lot of steric space among the three-dimensional cross-linked network system to provide a wonderful opportunity for the growth of nanoparticles within the gel matrix. In the present case, the RGO/silver nanoparticles composite hydrogels were obtained after the co-reduction of silver ions and GO to form RGO-based silver nanoparticles-containing composite hydrogel in the presence of vitamin C. The photos of GO/PEI gels, RGO/PEI gels, and silver nanoparticles-containing RGO/PEI gels were shown in Fig. 1C, respectively. The samples possess good gelation properties. The RGO hydrogen and silver nanoparticles within the RGO-based gel were characterized by scanning electron microscopy (SEM), transmission electron microscopy (TEM), X-ray diffraction (XRD) analysis, atomic force microscopy (AFM), and Raman spectroscopy.

SEM and TEM studies were utilized to examine the morphologies of RGO-based gel and silver nanoparticles containing RGO based hybrid gels. Both SEM and TEM images of the RGO hydrogel (Fig. 1A,D) show the GO sheets were cross-linked in the porous PEI networks. To compare with RGO-based gels, the SEM and TEM images of silver nanoparticles-containing RGO-based composite gels (shown in Fig. 1B,E) reveal that the as-formed silver nanoparticles were homogeneously and uniformly deposied on the surface of the RGO nanosheets to obtain a gel-based nanocomposite system. In addition, the detailed chemical analyses of RGO/PEI/Ag hydrogel were carried out using EDXS, as shown in Fig. 1F. The chemical signatures thus obtained were identical within experimental accuracy, and essentially Ag, Cu, and C elements were observed. The Cu peaks originated from the TEM grid. It was confirmed by EDXS that we have obtained the silver nanostructures in the presence of vitamin C. The diameters of Ag nanoparticles were determined from the SEM and TEM images, which are mainly in the range of 60–70 nm for Ag nanoparticles. The uniform and homogenous decoration of metal nanoparticle on the reduced grapheme oxide nanosheets can be mainly attributed to *in situ* reduction of metal salts within the gel matrix.

**Figure 2:**
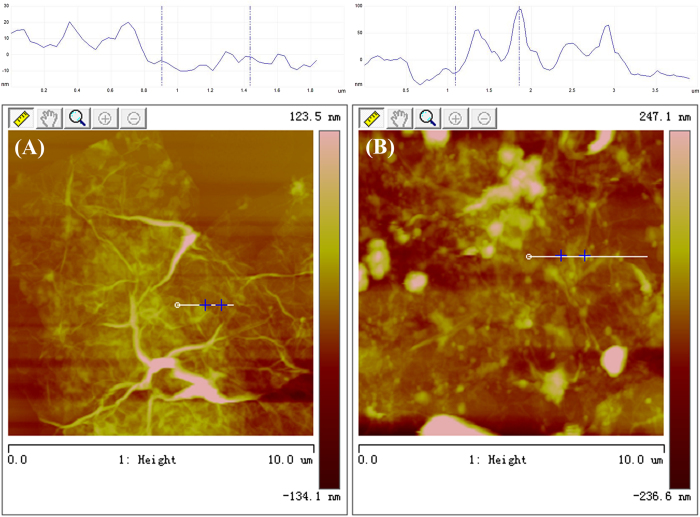
AFM images with section analysis of lyophilized RGO/PEI gels (**A**) and RGO/PEI/Ag gels (**B**).

AFM study of the RGO/PEI hydrogel and RGO/PEI/Ag hydrogel (Fig. 2) clearly exhibited the formation of 3D structured sheets. These sheets are cross-linked with one another to form a large 3D network like structure in the presence of PEI. The driving forces for forming the large 3D network-like structure are weak interactions, such as hydrogen bonding and π-π stacking^31^,^32^. The AFM image of the RGO/PEI/Ag hydrogel in Fig. 2B shows that the as-formed silver nanoparticles are homogeneously and uniformly modified on the surface of RGO nanosheets to obtain a gel based nanocomposite system.

**Figure 3:**
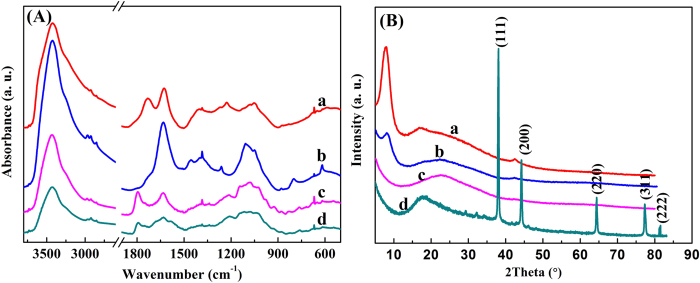
IR spectra (**A**) and XRD patterns (**B**) of lyophilized samples: a, GO sheet; b, GO/PEI gel; c, RGO/PEI gel; d, RGO/PEI/Ag gel.

The FT-IR spectra of GO, GO/PEI hydrogel, RGO/PEI hydrogel, and RGO/PEI/Ag hydrogel were demonstrated in Fig. 3A. In the spectrum of GO, the peak at 3424 cm^−1^ was correlated to the -OH vibration stretching. Figure 3A also shows the IR bands of carboxyl C = O (1724 cm^−1^), epoxy C-O (1226 cm^−1^) and alkoxy C-O (1050 cm^−1^) groups situated at the edges of the GO nanosheets^33^,^34^,^35^. In the spectrum of GO/PEI gel, a peak observed at 1645 cm^−1^ corresponds to the -NH stretching of PEI. In the spectra of RGO/PEI gel and RGO/PEI/Ag gel, the peaks of -NH stretching of PEI, carboxyl C = O and C-O, as well as alkoxy C-O can be found, while the peak of epoxy C-O at 1226 cm^−1^ disappeared. The FT-IR results clearly indicated that GO has been reduced into RGO, the obvious amide absorption peak could be observed at 1640 and 1385 cm^−1^. The analysis of the FT-IR spectra confirms the successfully preparation of the RGO/PEI/Ag composite hydrogel.

In addition, the strong affiliation between PEI and GO was also evidenced by X-ray diffraction study. Figure 3B shows the diffraction patterns for the GO, the lyophilized GO/PEI hydrogels, the lyophilized RGO/PEI hydrogels and the lyophilized RGO/PEI/Ag hydrogels. The 2θ values were observed at 11.3° (GO), and 8.9° (GO/PEI hydrogel), suggesting the increase in the spacing between GO layers with the addition of the PEI content in the hydrogels. The XRD pattern of the dried RGO hydrogels vividly shows that the appearance of a very broad peak centered at 2θ = 22.4° and the complete disappearance of diffraction peak at 2θ = 11.3° (for GO gel). This clearly indicates the almost complete reduction of GO sheets to reduced graphene oxide (RGO) nanosheets in RGO gel with the removal of oxygen-containing functionalized groups in GO^36^. For the silver nanoparticles-containing RGO-based composite dried hydrogel, there are diffraction peaks at 2θ = 17.72°, 38.12°, 44.28°, 64.43°, 77.47°, and 82.54°. The first diffraction peak at 2θ = 17.74° suggests the reduction of GO sheet to form RGO sheet in the gel. Other diffraction peaks at 2θ = 38.12°, 44.28°, 64.43°, 77.47°, and 82.54° are consistent with those for Ag standard card (JCPDS card no. 04-0783, space group Fm-3m(225)) and they correspond to the (111), (200), (220), (311), and (222) Miller indices of Ag, respectively.

**Figure 4:**
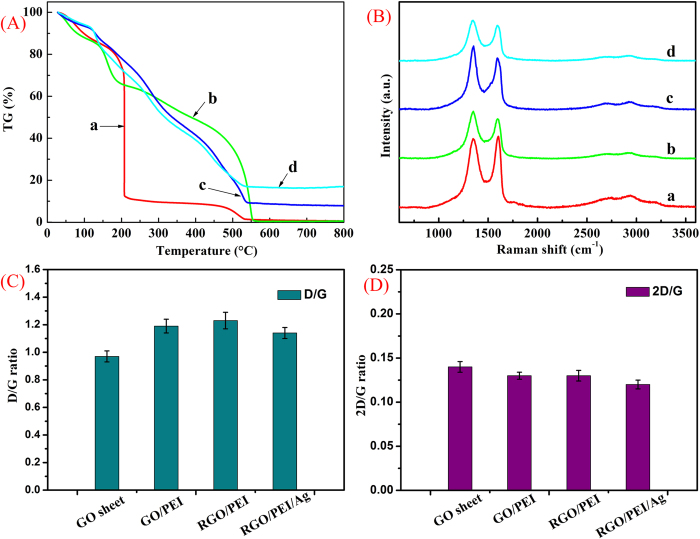
TG (**A**) and Raman spectroscopy (**B**) of lyophilized samples: a, GO sheet; b, GO/PEI gel; c, RGO/PEI gel; d, RGO/PEI/Ag gel. (**C**) and (**D**) present D/G and 2D/G ratios of the Raman spectra shown in (**B**), respectively.

Figure 4A illustrates the thermograms of GO and GO-based composite hydrogels. According to TG results, GO/PEI hydrogels, RGO/PEI hydrogels, and RGO/PEI/Ag hydrogel show a higher thermal stability compared with GO, which could be attributed to the higher cross-linking within the networks. In fact, the addition of low-content PEI can greatly increase the thermal stability of hydrogels, suggesting strong interactions between GO sheets and PEI, even in such a water-abundant hydrogel. The two mass loss peaks at about 200 and 500 °C of the hydrogels originate from the pyrolysis of the oxygen containing functional groups and PEI moieties, respectively. When the temperature is higher than 500 °C, the qualities of the RGO-based hydrogels are not changed, and the quality of RGO/PEI/Ag hydrogel is essentially higher than RGO/PEI hydrogel. It was reported that some GO composites showed different weight retention value at high temperature, probably due to the structural changes from the existence of carbon net-compounds assembly structure in the composites^37^,^38^,^39^. In the present case, the as-formed silver nanoparticles in the composite hydrogels enhance the thermal stability of materials to a certain extent.

Raman spectroscopy provides a useful tool to characterize the carbon-based materials^40^, as shown in Fig. 4B. Three famous bands of graphene sheets in Raman spectra appeare, including the G band (1601 cm^−1^) originated from the first-order scattering of the E_2_g phonons of the sp^2^-hybridized carbon atoms, the D band (1351 cm^−1^) caused by a breathing mode of κ-point phonons of A_1_g symmetry of the defects involved in the sp^3^-hybridized carbon bonds such as hydroxyl and/or epoxide bonds^41^, and the 2D band (2692 cm^−1^) which is much sensitive to stacking of graphene sheets^42^. It is established that the G and 2D bands of single-layer graphene sheets usually locate at 1585 and 2679 cm^−1^, while for multi-layer graphene sheets (including 2–6 layers), the positions of the G and 2D bands shift into lower and higher wavenumbers, respectively^43^,^44^. Furthermore, the 2D/G ratios of single-, double-, triple- and multi- (>4) layer graphene sheets are typically >1.6, 0.8, 0.30 and 0.07, respectively^45^,^46^. For example, O. Akhavan *et al.* achieved excellent research work and reported successfully the 2D/G ratios of the single and bilayer GO sheets in the range of 1.53–1.68 and 0.82–0.89, respectively^47^. In our present work, the 2D/G ratios of the GO sheets and three different composite gels showed the values in the range of 0.12–0.14 (seen in Fig. 4D), suggesting the multilayer nature in present prepared graphene sheets. In addition, due to the origination of the G and D bands, the G/D peak intensity ratio is known as a measure of the sp^2^ domain size of graphene sheets containing sp^3^ and sp^2^ bonds. In our present work, it was found that by forming the composite gels, the D/G ratio shifted from 0.97 to the range of 1.14–1.23, as shown in Fig. 4C. This result can be attributed to the successful cross-linking of GO in the hydrogels networks and the absence of the C-N bonds formed on surface of the RGO sheets.

**Figure 5:**
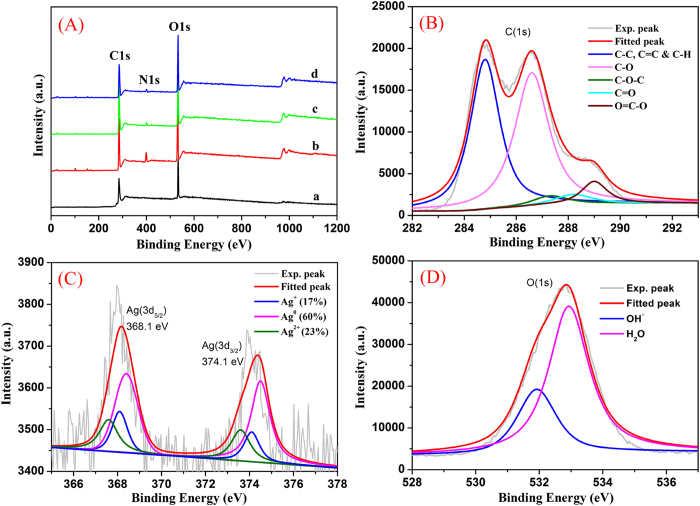
Survey XPS spectra (**A**) of lyophilized samples: a, GO sheet; b, GO/PEI gel; c, RGO/PEI gel; d, RGO/PEI/Ag gel. Deconvolution of XPS peaks of the RGO/PEI/Ag nanocomposite: **B**, **C**(1s); **C**, Ag(3d); **D**, O(1s).

Since the obtained RGO/PEI/Ag nanocomposites were prepared to apply in the photocatalytic purposes, their surface analysis and interfacial composition are of great importance. Firstly, the survey XPS spectra of all lyophilized samples in Fig. 5A showed the characteristic peaks, such as C(1s), N(1s), and O(1s). In addition, we obtained the relative elemental composition and calculated the O/C ratios of all lyophilized samples (GO sheet, 37.26%; GO/PEI gel, 36.08%; RGO/PEI gel, 32.84%; RGO/PEI/Ag gel, 33.46%), respectively, which suggested the decrement of oxygen element from GO to RGO process. Moreover, the deconvolution C(1s), Ag(3d), and O(1s) of XPS peaks for the RGO/PEI/Ag nanocomposite were demonstrated. Figure 5B shows XPS peak deconvolution of C(1s) core levels of the RGO/PEI/Ag nanocomposite. The peak centered at 284.8 eV was attributed to the C-C, C = C and C-H bonds. The other deconvoluted peaks located at the binding energies of 286.7, 287.4, 288.2 and 289.2 eV were assigned to the C-OH, C-O-C, C = O, and O = C-OH oxygen-containing bonds, respectively^48^. Figure 5C presents the high-resolution XPS spectrum of Ag(3d) core level in the RGO/PEI/Ag gel. The Ag(3d_5/2_) and Ag(3d_3/2_) peaks were found at binding energies of 368.1 and 374.1 eV, respectively. Moreover, the slitting of the 3d doublet of Ag is 6.0 eV, indicating the formation of metallic silvers in the RGO/PEI/Ag composite^49^. To further understand the chemical state of the silvers, a detailed deconvolution of the Ag(3d) peak was also performed. The binding energy of Ag(3d_5/2_) core level for Ag, Ag_2_O and AgO is 368.4, 368.1 and 367.6 eV, respectively. Hence, the Ag(3d_5/2_) peak was deconvoluted into three Gaussian components with identical FWHM after a Shirley background subtraction. Based on the deconvolution analysis, we found that about 60% of the silvers were in the Ag^0^ (metallic) state, while about 17% and 23% of them were in Ag^+^ and Ag^2+^ chemical states, respectively. In addition, the O(1s) photoelectron peak of the RGO/PEI/Ag nanocomposite was shown in Fig. 5D. This peak can be deconvoluted into two Gaussian components with identical FWHM after a Shirley background subtraction. The first component at 532.0 eV can be corresponded to the oxygen of surface OH^–^ bound in the nanocomposite, in which the photogeneration of electron-hole changes into OH· free radicals that is of benefit to photocatalysis processes^50^. The second deconvoluted O(1s) peak at 533.0 eV was attributed to the oxygen in water molecules existed in the nanostructure or adsorbed on the GO surface. This means that the surface of RGO/PEI/Ag nanocomposite was still porous, which can be an advantage for surface photocatalytic processes.

**Figure 6:**
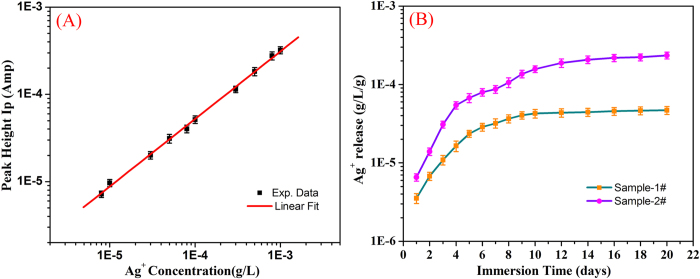
Standard curve of silver ions for calibration of the ion current (**A**) and the silver ion release curves of RGO/PEI/Ag nanocomposite after measurements in HNO_3_ solution (**B**).

The silver ion release behavior from the RGO/PEI/Ag nanocomposites was also investigated. Figure 6 shows the time-dependent patterns for silver ion release. It clearly indicated that the quantities of released silver ion increased sharply in initial 6 days. After that, the release rate of silver ion is relatively slow, especially after 12 days. Such release behavior of silver ions from RGO/PEI/Ag nanocomposite is similar to the reports about the Ag/a-TiO_2_ thin films and other silver-containing composite systems^51^,^52^,^53^,^54^,^55^. These results reveal that a slow release mechanism of silver ions for the mesoporous RGO/PEI/Ag nanocomposites comparing with that for bulk matrices. The amount of released silver ions was measured to be 4.696 × 10^−5^ and 2.348 × 10^−4^ g/L/g after 20 days for sample-1# and sample-2#, respectively.

**Figure 7:**
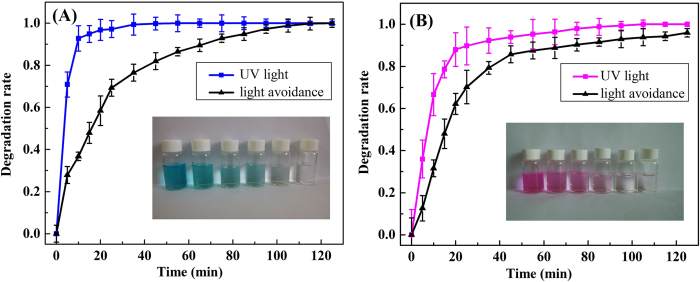
Photocatalytic properties of RGO/PEI/Ag gel on MB (**A**) and RhB (**B**) solution, respectively. The inserted photos are dye solutions acquired for the supernatant liquids collected at different time intervals during photocatalytic experiment.

The catalytic property of the as-formed Ag nanoparticle-containing RGO based composite hydrogel matrix was also investigated for the photocatalytic degradation of RhB and MB solutions. The dye degradation capacity was evaluated by placing the as-prepared RGO/PEI/Ag hydrogels in RhB and MB aqueous solutions. Figure 7 shows the calculated dye degradation rate versus time plots for both RhB and MB solutions in the presence of Ag nanoparticles-containing RGO-based composite hydrogel as a catalyst. The dye degradation rates can reach nearly 100% for RhB within approximately 70 mins, but for MB with approximately 30 mins, suggesting the high efficiency of the as-prepared RGO/PEI/Ag hydrogels as dye catalysts. In contrast, without photo irradiation, the degradation performance of RGO/PEI/Ag hydrogels was significantly reduced.

**Figure 8:**
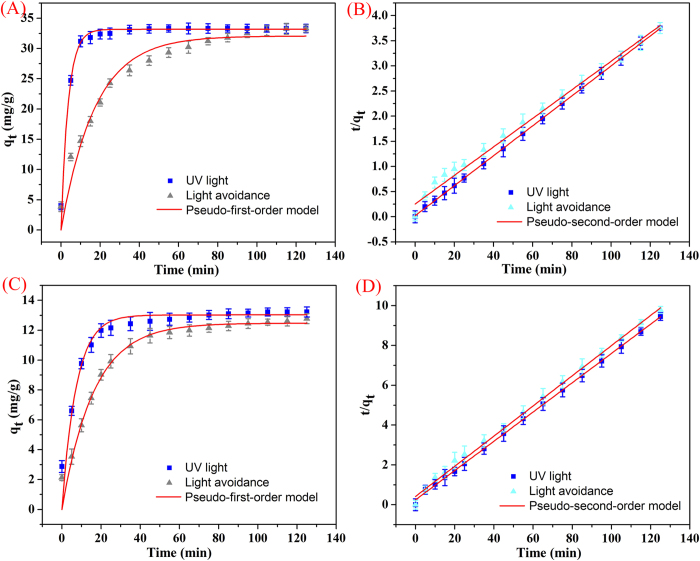
Degradation kinetics curves of as-prepared RGO/PEI/Ag nanocomposites on MB (**A**,**B**) and RhB (**C**,**D**) at 298 K.

Degradation kinetic experiments of the as-prepared RGO/PEI/Ag nanocomposites on MB and RhB were performed, and the results were shown in Fig. 8. The nanocomposites exhibit a rapid adsorption process, with equilibrium times of approximately 80 and 100 minds for MB and RhB, respectively. The slightly prolonged kinetic behavior observed for RhB may be ascribed to the different absorbed process from dye incorporation to GO surface. A 100 min equilibrium time is sufficient for efficient photocatalytic applications. Such kinetic behavior can also be associated with the unique nanocomposites structure, i.e., the large three-dimensional network-like nanostructure cross-linked with PEI by electrostatic attractions and hydrogen bonding, and highly dispersed Ag nanoparticles modified on the surface of RGO nanosheets as the photocatalytic active sites. In addition, classical kinetic models were employed to describe the above degradation data as follows:

The pseudo-first-order model can be described by equation (1):


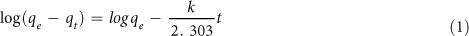


The pseudo-second-order model can be described by equation (2):


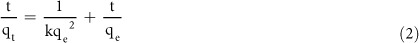


where q_e_ and q_t_ represent the amount of dye degraded (mg/g) at equilibrium and time t, respectively, and the k_1_ and k_2_ values are the kinetic rate constants. The kinetic data (Table 1) can be accurately described by the pseudo-second-order model with a high correlation coefficient (R^2^ > 0.992).

**Figure 9:**
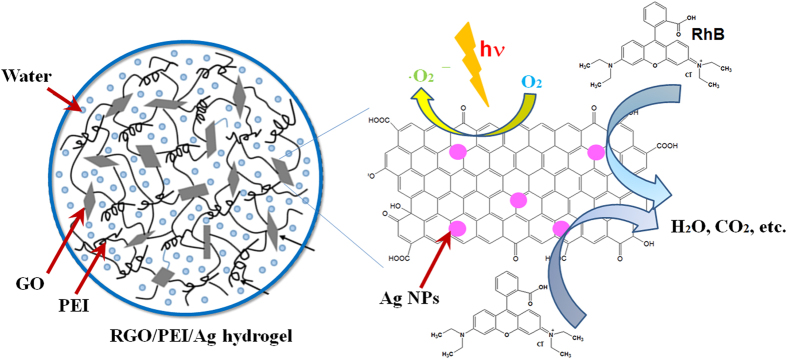
Proposed scheme of photocatalytic degradation of RGO/PEI/Ag gel on dye solution.

The charge transfer mechanism that occurs in the RGO/PEI/Ag nanocomposite during photocatalytic process is shown in Fig. 9. During the photocatalysis reaction, dye molecules could be transferred from the solution to the composite’s surface and be adsorbed with offset face-to-face orientation via π–π conjugation between RhB (and MB) and aromatic regions of the graphene^56^. When UV irradiation was applied to the surface of RGO/PEI/Ag nanocomposite, the photo-excited electrons can quickly inject to graphene sheets and then reacted with adsorbed O_2_ molecules on the graphene to produce O_2_^−^ and/or O_2_^2−^ radicals^57^. In this way, the prepared composite could generate more electrons and holes, and generate more superoxide anions and/or peroxide species^58^. As the result of production of above, dyes are fragmented to H_2_O, CO_2_ and other mineralization. Due to the process of electron transfer, charge recombination is suppressed in RGO/PEI/Ag composite and hence largely enhances the efficiency of photocatalytic properties.

In summary, we have demonstrated the facile preparation and dye degradation capacity of RGO/PEI/Ag hydrogels. The PEI was chosen for its abundant amine groups that can form hydrogen bonding with GO. Both the SEM and AFM studies clearly showed that the GO sheets were successfully cross-linked in the PEI network, and the silver nanoparticles are homogeneously and uniformly fabricated on the surface of the RGO nanosheets to create a gel based nanocomposite system. Meanwhile, the Raman and XPS spectra suggested that the structural features of GO sheets remain largely unchanged prior and post gelation. The as-prepared RGO/PEI/Ag hydrogels exhibited good photocatalytic rates for both RhB and MB. The current study provides further insight into the applications of noble metal nanoparticles-containing RGO based functional composite hydrogels as dye catalysts for waste water treatment.

## Methods

The experimental used materials, polyethyleneimine (PEI, M_w_ = 500 g·mol^−1^, Aladdin Reagent, Shanghai, China), silver acetate (CH_3_COOAg, Aladdin Reagent, Shanghai, China), rhodamine B (RhB, Tianjin KaiTong chemical reagent co., LTD) and methylene blue (MB, Tianjin KaiTong chemical reagent co., LTD) was used as received. Sulfuric acid (H_2_SO_4_, 98%), graphite powder (99.85% purity), vitamin C (ascorbic acid), potassium permanganate (KMnO_4_), potassium nitrate (KNO_3_), hydrogen peroxide (H_2_O_2_, 30%, w/w), hydrochloric acid (HCl), were of analytical reagent grade (AR) and were used without further purification. All aqueous solutions were prepared with deionized (DI) water in whole experiment process.

Graphene oxide was prepared from graphite powder (325 mesh, 99%, Alfa Aesar Chemicals) by a modified Hummers method^59^. In a typical procedure, 50 ml of H_2_SO_4_ (98%) was added into a 500 ml flask and cooled by immersion in an ice bath. Then, 1.5 g KNO_3_ was added into the sulfuric acid solution under vigorous stirring to avoid agglomeration. After potassium nitrate was well dispersed, 1 g graphite powder was added along the beaker wall. Then 8 g KMnO_4_ was added gradually under stirring, the rate of addition was carefully controlled to keep the reaction temperature below 10 °C. After 2 h, the ice bath was removed and the mixture was maintained at 35 °C by thermostatic oil bath for 6 h. After the reaction, 200 ml of H_2_O was slowly added under vigorous stirring, the rate of addition was 3–5 drops per second, successively. Then the reaction temperature rapidly increased to 80 °C. After 30 min, the mixture was further treated with 200 ml of H_2_O and 20 ml of 30% H_2_O_2_. For purification, the mixture was filtered and washed with 5% HCl aqueous to remove metal ions and through the centrifugal cleaning with DI water several times, the pH of the supernatant was neutral. After centrifugation and freeze drying in low temperature (−50 °C), graphene oxide was obtained.

To confirm the approach is a universal protocol, GO hydrogels were prepared using polyethylene imine including RGO/PEI hydrogel and RGO/PE/Ag hydrogel. At first, a stock solution (108 mg/mL) of vitamin C (ascorbic acid) has been prepared by dissolving it in deionized water and then 0.2 ml of this solution has been added into the 0.8 ml aqueous dispersion of GO (9 mg/mL). The mixture in a glass vial has been stirred for several minutes. After that, 0.2 ml of polyethylene imine (5.4 mg/mL) has been dispersed in the mixed solution and the final mixture has sonicated continuously for about 10 minutes followed by heating at 90 °C for 10 minutes. In the process of heating, the GO gel has been transformed gradually to a reduced graphene oxide (RGO)-based hydrogel within a few minutes. The RGO gel has started to shrink with the increasing time. The gel has floated into the glass vial after a few minutes. Eventually, a reduced graphene oxide-based hydrogel has been obtained.

Similar to the above synthesis method, RGO/PEI/Ag hydrogel has been prepared. At first, 50 μL of aqueous solution of metal salt (CH3COOAg) (8 mg/mL) has been added into 0.8 ml of aqueous dispersion of GO (12 mg/mL) and has been stirred and sonicated occasionally. After that, a stock solution (240 mg/mL) of vitamin C has been prepared by dissolving it in deionized water and then 0.2 mL of this solution has been added into the mixed solution, followed by the addition of 0.2 mL of polyethylene imine (7.2 mg/mL). This mixture has been sonicated well. It was then heated well at 90 °C for 10 min. This has resulted in the formation of the corresponding metal nanoparticle containing rGO-based hybrid hydrogels. In this study, we have believed that metal ions have been reduced *in situ* by the ascorbic acid within the hydrogel matrix.

For silver ion release measurements from the prepared nanocomposites, the process was carried out concomitantly with the so-called “Erlanger silver catheter” as described^60^. At first, the samples of the lyophilized RGO/PEI/Ag nanocomposite (5 mg abbreviated as sample-1#, 20 mg abbreviated as sample-2#) were immersed in a flask containing 100 ml HNO_3_ solution (0.1 M) at room temperature. Then, the concentrations of Ag^+^ released were measured by anodic stripping voltammetry (ASV) of 1 ml of the prepared solution. The ASV measurements for silver ions release were carried out on a CHI660b electrochemical analyzer, using a conventional three-electrode electrochemical cell containing a glass carbon electrode and a Pt wire as the working and counter electrodes, respectively. In addition, Ag/AgCl electrode with saturated KCl solution was employed as reference electrode. The discharge potential was −0.55 V, and the stripping scan was carried out in the potential range of 0–0.6 V with a scan rate of 100 mV/s. It should also to be noted that the current of released silver ions was calibrated by measuring the currents of AgNO_3_ solutions with various concentrations in our experimental conditions.

For catalytic experiment, the reaction was processing at room temperature. A small amount of freshly prepared RGO/PEI/Ag hydrogel catalyst was added in 100 mL of a certain concentration of RhB (4 mg/L) and MB solution (10 mg/L). The magnetic stirring made the catalyst dispersed evenly, and then the mixed solution stirred under the condition of the dark for 30 min to reach adsorption equilibrium. After that, the mixed solution was irradiated by the high pressure mercury lamp (365 nm, 100 W) with the light source about 15 cm distances from liquid surface. When the UV lamp was preheated about five minutes, the reaction conducted, the concentrations of the organic dyes were determined at different time intervals. The samples were separated by centrifugation and the supernatant solutions were analyzed by 752 UV-vis spectrometer (Sunny Hengping scientific instrument Co., Ltd, Shanghai) at the wavelength of 632 nm (MB) and 554 nm (RhB) with the pre-established calibration curves respectively. The calculation of dye degradation rate (K) was as follows:


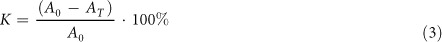


Where K is the degradation rate; A_O_ is the absorbance of the original solution; A_T_ is the absorbance of the solution at any time.

The morphology of GO, lyophilized RGO/PEI hydrogels and lyophilized RGO/PEI /Ag hydrogels were characterized by using both a field-emission scanning electron microscopy (FE-SEM, S-4800II, Hitachi, Japan) with the accelerating voltage of 5–15 kV and a transmission electron microscopy (TEM, HT7700, Hitachi High-Technologies Corporation). The chemical composition of the samples was characterized by energy-dispersive X-ray spectroscopy (EDXS). EDXS analysis was typically performed at an accelerating voltage of 200 kV, using an Oxford Link-ISIS X-ray EDXS microanalysis system attached to TEM. FTIR spectra were recorded on a Fourier infrared spectroscopy (Thermo Nicolet Corporation) with milled dried gels by the conventional KBr disk tablet method. Thermogravimetry-differential scanning calorimetry (TG-DSC) analyses of the samples were conducted in air condition by using NETZSCH STA 409 PC Luxxsi multaneous thermal analyzer (Netzsch Instruments Manufacturing Co., Ltd., Germany). X-ray diffraction study was carried out by using an X-ray diffractometer (SMART LAB, Rigaku) equipped with a conventional Cu Kα X-ray radiation (λ = 1.54 Å) source and a Bragg diffraction setup. Raman spectroscopy was performed using a Horiba Jobin Yvon Xplora PLUS confocal Raman microscope equipped with a motorized sample stage. The wavelength of the excitation laser was 532 nm and the power of the laser was kept below 1 mW without noticeable sample heating. The intensity of a Raman peak was extracted from the maximum value after baseline subtraction over corresponding spectral range. Atomic force microscopy (AFM) images were measured by using a Nanoscope model Multimode 8 Scanning Probe Microscope (Veeco Instrument, USA) with silicon cantilever probes. X-ray photoelectron spectroscopy (XPS) was performed on the Thermo Scientific ESCALab 250Xi using 200 W monochromated Al Kα radiation. The 500 μm X-ray spot was used for XPS analysis. The base pressure in the analysis chamber was about 3 × 10^−10^ mbar. Typically the hydrocarbon C1s line at 284.8 eV from adventitious carbon is used for energy referencing. Both survey scans and individual high-resolution scans for Ag(3d), O(1s) and C(1s) peaks were recorded. ASV measurements for silver ions release were carried out on a CHI660b electrochemical analyzer (CHI Inc., USA), using a conventional three-electrode electrochemical cell. The discharge potential was −0.55 V with a deposition time of 1000 s. The stripping scan was carried out ranged from 0 to 0.6 V with a scan rate of 100 mV s^−1^.

## Additional Information

**How to cite this article**: Jiao, T. *et al.* Reduced Graphene Oxide-Based Silver Nanoparticle-Containing Composite Hydrogel as Highly Efficient Dye Catalysts for Wastewater Treatment. *Sci. Rep.* **5**, 11873; doi: 10.1038/srep11873 (2015).

## Figures and Tables

**Table 1:** Kinetic parameters of RGO/PEI/Ag nanocomposite for MB and RhB degradations at 298 K (experimental data from Fig. 8).

	**Pseudo-first-order model**			**Pseudo-second-order model**		
	**q_e_ (mg/g)**	**R^2^**	**K_1_ (min^−1^)**	**q_e_ (mg/g)**	**R^2^**	**K_2_ (g/min·h)**
MB						
UV light	32.0744	0.95487	0.05758	35.249	0.99209	0.00316
Light avoidance	33.1817	0.97796	0.27175	33.591	0.99991	0.03858
RhB						
UV light	12.4753	0.94737	0.06164	13.270	0.99779	0.00118
Light avoidance	13.0280	0.93771	0.13626	13.515	0.99934	0.02640
